# Rapid and Accurate Detection of *Escherichia coli* and *Klebsiella pneumoniae* Strains Susceptible/Resistant to Cotrimoxazole through Evaluation of Cell Elongation

**DOI:** 10.3390/antibiotics10060720

**Published:** 2021-06-15

**Authors:** Isidoro López, Fátima Otero, Rebeca Guillén, María del Carmen Fernández, Germán Bou, Jaime Gosálvez, José Luis Fernández

**Affiliations:** 1 Genetics Unit, INIBIC-Complejo Hospitalario Universitario A Coruña (CHUAC), 15006 A Coruña, Spain; isidoro.lopez.baltar@sergas.es (I.L.); fatima.otero.farina@sergas.es (F.O.); rebeca.maria.guillen.fajardo@sergas.es (R.G.); 2 Molecular Genetics and Radiobiology Laboratory, Centro Oncológico de Galicia, 15009 A Coruña, Spain; 3 Microbiology Division, INIBIC-Complejo Hospitalario Universitario A Coruña (CHUAC), 15006 A Coruña, Spain; MA.Carmen.Fernandez.Lopez@sergas.es (M.d.C.F.); German.Bou.Arevalo@sergas.es (G.B.); 4 Genetics Unit, Facultad de Biología, Universidad Autónoma de Madrid, 28049 Madrid, Spain; jaime.gosalvez@uam.es

**Keywords:** cotrimoxazole, trimethoprim-sulfamethoxazole, antibiotic resistance, cell length, rapid assay, Gram-negative

## Abstract

Trimethoprim-sulfamethoxazole is a well-known antibiotic that inhibits folic acid synthesis, a topic of renewed interest. Since resistant strains are increasingly more common, an early and accurate discrimination of susceptibility may assure confident therapy. Two morphological assays were performed in *Escherichia coli* (*n* = 50; 27 non-susceptible) and *Klebsiella pneumoniae* (*n* = 52; 18 non-susceptible). First, the strains were incubated with the CLSI breakpoint of cotrimoxazole for 150 min, which induced cell lengthening in the susceptible strains. Second, the bacteria were incubated with mitomycin C (MMC) (0.5 mg/L) for 120 min to induce a SOS-linked cell enlargement higher than that obtained by cotrimoxazole alone. When cotrimoxazole was added 30 min before MMC, the inhibition of folic acid synthesis in the susceptible strain resulted in the suppression of MMC-induced extra elongation. In the non-susceptible strains, folic acid synthesis continued despite the antibiotic, so that the MMC-induced extra cell lengthening could not be impeded. Whereas the first assay resulted in five false negatives and four false positives of resistance, the results of the second assay matched those of the conventional antibiogram. This simple morphological procedure is performed in 2 h and 45 min and may allow a rapid selection of useful and relatively inexpensive therapy, thereby preserving the newer broad-spectrum antibiotics.

## 1. Introduction

Cotrimoxazole, the combination of trimethoprim and sulfamethoxazole, was introduced in the late 1960s due to their synergistic activity to block bacterial folic acid synthesis [1,2]. Sulfamethoxazole is a sulfonamide, structural analog of para-aminobenzoic acid that competitively inhibits dihydropteroate synthase, an enzyme that catalyzes the formation of dihydrofolate from para-aminobenzoic acid and pteridine. Trimethoprim is a diaminopyrimidine antibiotic, structural analog of the pteridine component of dihydrofolate, behaving as a competitive inhibitor of dihydrofolate reductase, thus blocking the reduction of dyhydrofolic acid to tetrahydrofolic acid [3]. Tetrahydrofolic acid is the physiologically active form of folic acid, being an essential cofactor for the synthesis of thymidine, purines and methionine. The depletion of tetrahydrofolate as a consequence of the sequential enzymatic inhibition in its metabolic pathway hinders bacterial DNA replication and transcription, as well as protein synthesis, leading to a bactericidal effect.

Cotrimoxazole is usually active against Gram-negative bacilli and Gram-positive cocci but, also, *Pneumocystis jirovecii* and *Toxoplasma gondii* infections [3,4]. Nevertheless, the prescription of cotrimoxazole has been progressively declining during the past decade due to the spread of strains expressing genotypic and phenotypic resistance. In fact, cotrimoxazole remains as a first option for the treatment of noncomplicated urinary tract infections and in diseases caused by *Stenotrophomonas maltophilia* [5,6]. It is also prescribed for the acute infectious exacerbation of chronic obstructive pulmonary disease, otitis media in pediatric population, traveler’s diarrhea, shigellosis, pneumocystis pneumonia and toxoplasmosis [3,4]. More recently, due to the global spread of multidrug-resistant (MDR) Gram-negative bacilli, interest in the use of cotrimoxazole has been renewed. This is a widely available and relatively inexpensive medication whose adequate prescription may help to prevent the advancement of dangerous resistant pathogens by the misuse of antibiotics and to preserve newer broad-spectrum antibiotics and last option antibacterial drugs [7,8,9]. Since cotrimoxazole resistant strains are usual, an accurate determination of the susceptibility or resistance should be performed [10]. It is evident that the earlier the testing, the more reliable and successful the therapy will be.

Trimethoprim has been shown to induce filamentation in the clinical strains of some Enterobacteriaceae, and it has been proposed as a rapid test for the discrimination of susceptibility or resistance [11]. In a previous report, we introduced a rapid assay for the identification of resistant strains of Gram-negative bacilli to antibiotic inhibitors of protein synthesis based on the simple evaluation of the bacterial cell length [12]. The cultures were incubated with mitomycin C (MMC), which induced significant cell enlargement as a rapid consequence of the triggering of the SOS response [13,14]. An addition of the antibiotic before MMC prevented the elongation when the strain was susceptible to the antibiotic, thereby inhibiting protein synthesis. However, cell enlargement was not prevented in the antibiotic-resistant strains where protein synthesis had not been successfully inhibited [12]. Here, we demonstrate that the MMC-induced elongation assay, in addition to the antibiotic inhibitors of protein synthesis, can also be successfully adapted for the rapid detection of susceptibility or resistance to cotrimoxazole in *Enterobacteriaceae Escherichia coli* and *Klebsiella pneumoniae*. The discriminative capacity of the MMC-induced elongation assay may be more accurate than the evaluation of filamentation induced by the antibiotic.

## 2. Results

### 2.1. Technical Assay

**Figure 1:**
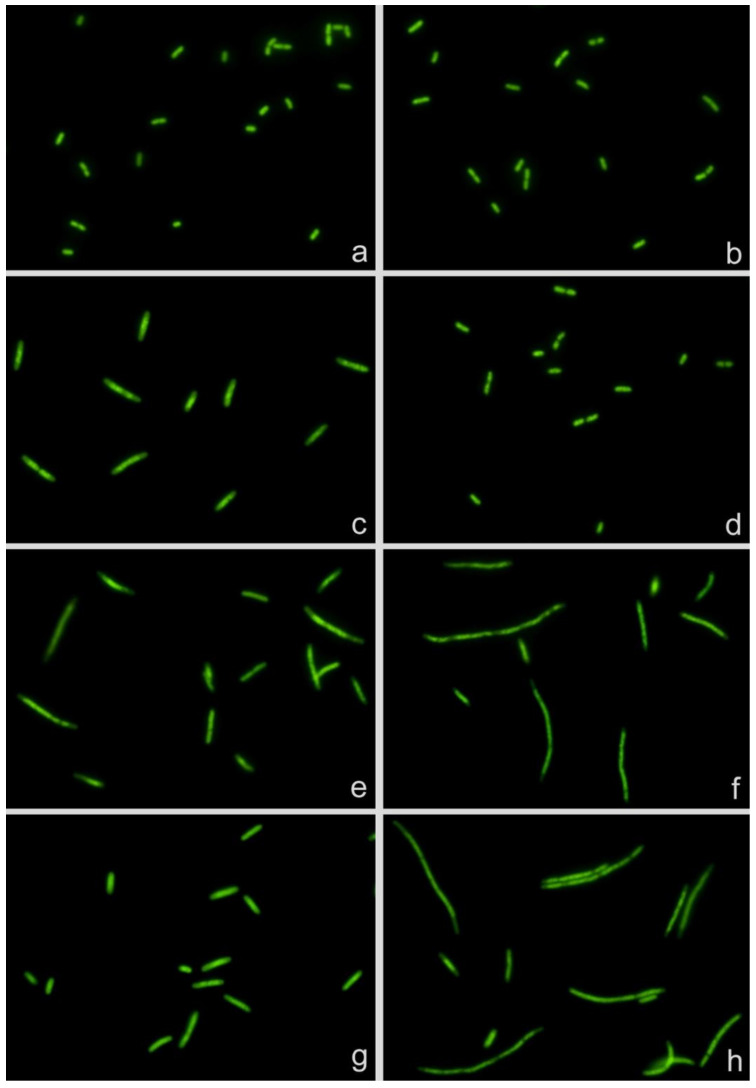
*Escherichia coli* strains susceptible (left, MIC: 0.125 mg/L) and resistant (right, MIC: >32 mg/L) to cotrimoxazole. (**a**,**b**) Control without antibiotic. (**c**,**d**) Incubated with cotrimoxazole, 2 mg/L. (**e**,**f**) Treated with mitomycin C (MMC), 0.5 mg/L. (**g**,**h**) Incubated with cotrimoxazole and MMC. MMC induces cell elongation in both strains (**e**,**f**). Cotrimoxazole induces cell lengthening and prevents MMC-induced cell enlargement in the susceptible strain (**c**,**g**) but not in the resistant strain (**d**,**h**).

The technical bases of the assay are presented in Figure 1. Two strains of *E. coli*, one susceptible (S) to cotrimoxazole (MIC: 0.125 mg/L) and one resistant (R) (MIC: >32 mg/L), were incubated with the CLSI (Clinical and Laboratory Standards Institute) breakpoint concentration of the antibiotic (2 mg/L) for 150 min, with MMC (0.5 mg/L) for 120 min and with cotrimoxazole 150 min and MMC added during last 120 min. Cotrimoxazole alone increased the cell length in the susceptible strain but not in the resistant strain (basal-S: 2.18 ± 0.39 μm; basal-R: 2.16 ± 0.46 μm; cotrimoxazole-S: 3.61 ± 1.19 μm; cotrimoxazole-R: 2.33 ± 0.37 μm; mean ± SD). MMC alone induced cell lengthening in both strains, higher than that produced by cotrimoxazole alone (MMC-S: 4.57 ± 2.18 μm; MMC-R: 6.49 ± 4.13 μm). When cotrimoxazole was added 30 min before MMC, the susceptible strain could not enlarge more than that achieved by cotrimoxazole alone, whereas the resistant strain increased the cell length similarly to that from the culture incubated with MMC alone (cotrimoxazole + MMC-S: 3.68 ± 1.35 μm; cotrimoxazole + MMC-R: 6.35 ± 4.10 μm). According to these results, two morphological parameters may provide information about the susceptibility or resistance to cotrimoxazole (1): cotrimoxazole-induced lengthening and (2) cotrimoxazole-induced suppression of MMC-linked elongation.

### 2.2. Dose Response

**Figure 2:**
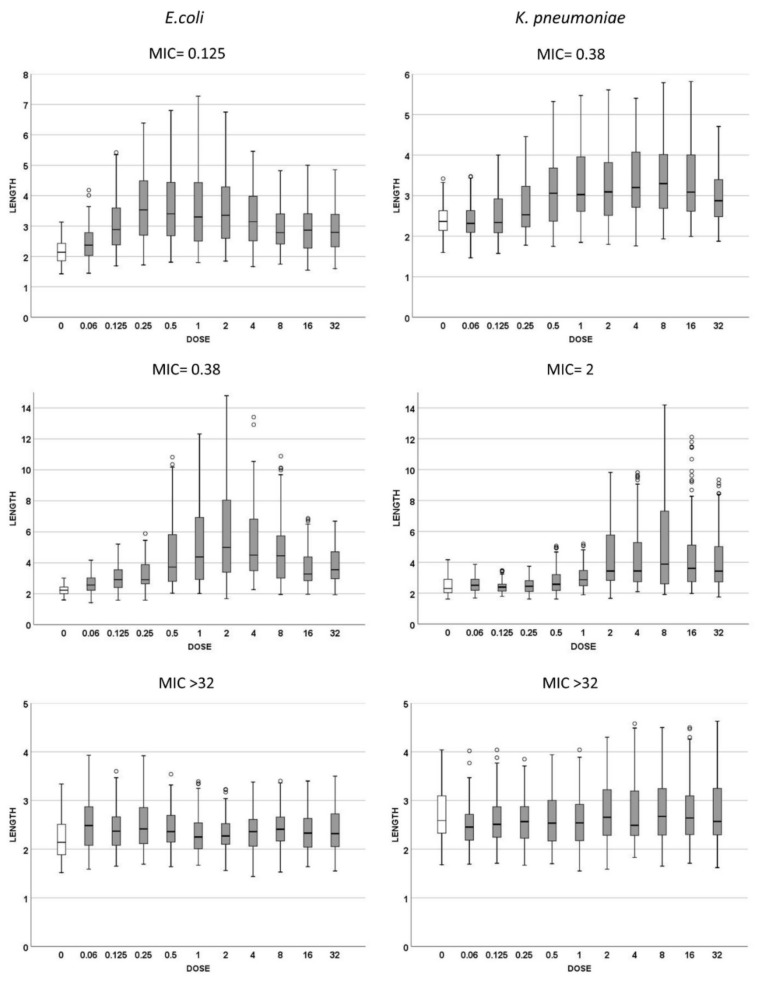
Cell length measures (μm) of Escherichia coli (left) and Klebsiella pneumoniae (right) strains with different MICs for cotrimoxazole. The first row corresponds to susceptible strains with MIC 0.125 mg/L for *E. coli* and 0.38 mg/L for *K. pneumoniae*; the second row represents the strains with MIC 0.38 mg/L for *E. coli* and 2 mg/L for *K. pneumoniae*; the third row represents the resistant strains, both with MIC > 32 mg/L. Each strain shows the cell length (vertical axis) measured in cultures incubated with increasing doses of cotrimoxazole, from 0 to 32 mg/L. The data are presented as box and whisker plots. The horizontal line in the box indicates the median, the lower line of the box is the first quartile, the upper line of the box is the third quartile and the whiskers (the end of the vertical lines) are the maximum and minimum values. Abnormal values are presented as dots outside the box.

**Figure 3:**
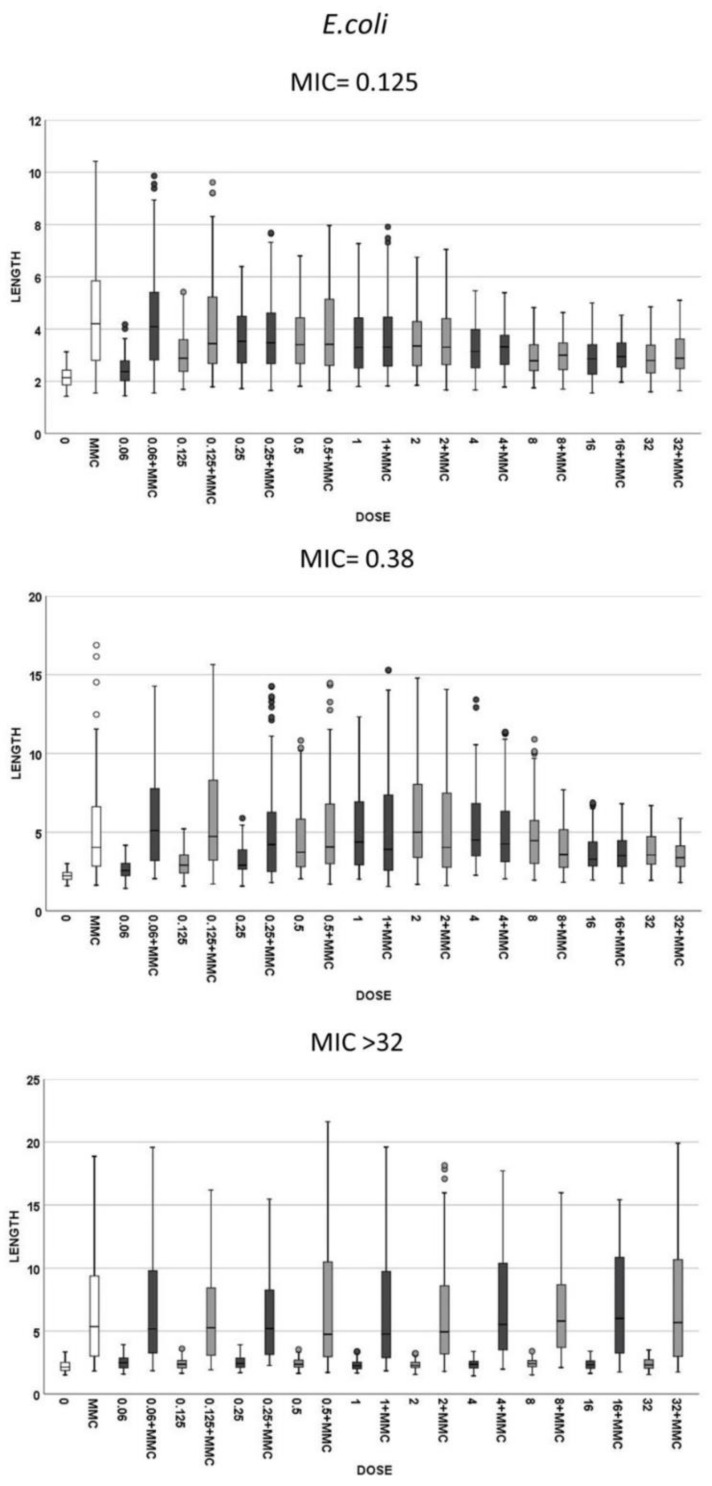
Cell length measures (µm) of *E. coli* in the same strains that were presented in Figure 2. The first and second graphics correspond to the strains susceptible to cotrimoxazole, with MIC 0.125 and 0.38 mg/L, respectively, and the third is a resistant strain with MIC > 32 mg/L. Each strain shows the cell length (vertical axis) in, from left to right (horizontal axis), control cultures (0), cultures incubated with 0.5-mg/L mitomycin C (MMC) and cultures incubated with increasing doses of cotrimoxazole alone, each paired with incubation of cotrimoxazole together with 0.5-mg/L MMC.

**Figure 4:**
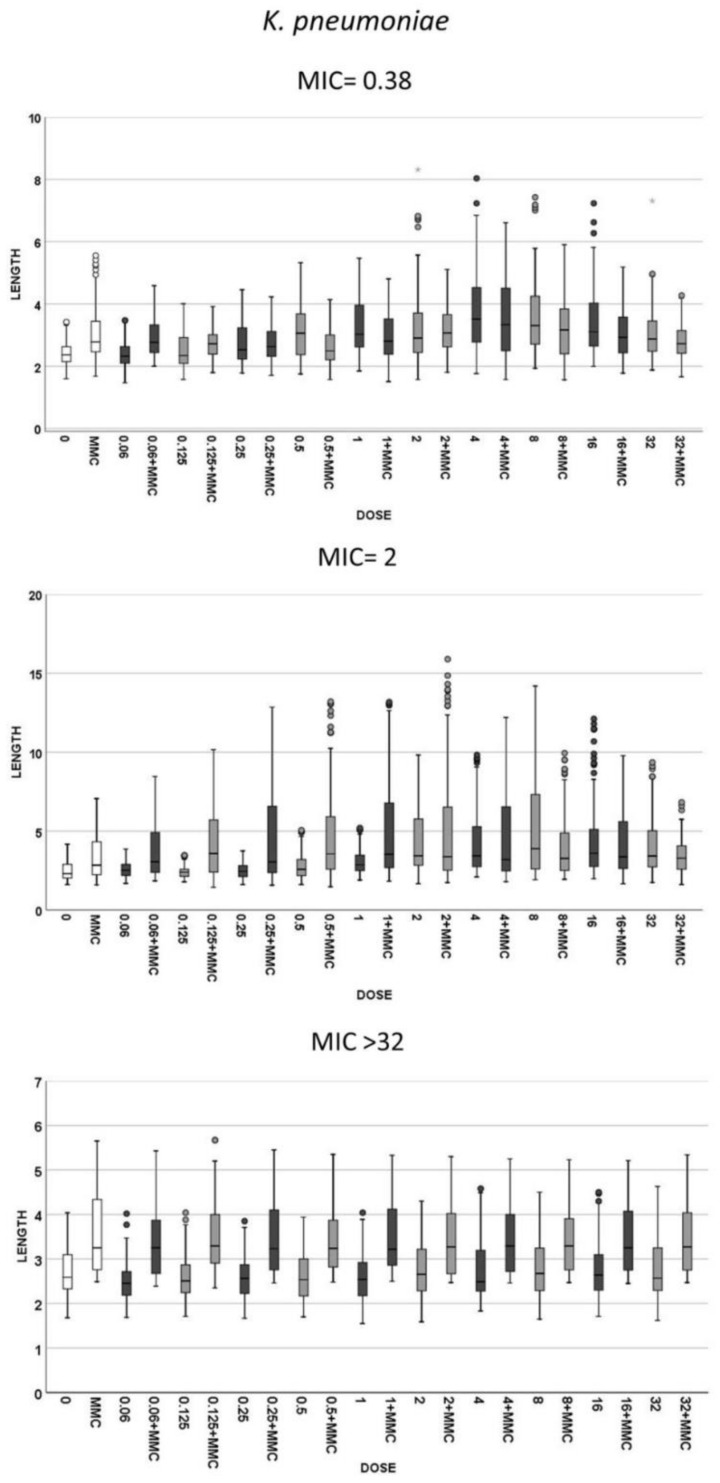
Cell length measures (µm) in the same strains of **K. pneumoniae** that were shown in Figure 2. The first image corresponds to a strain susceptible to cotrimoxazole, with MIC 0.38 mg/L, the second graphic is a strain with MIC 2 mg/L and third image is a resistant strain with MIC ≥ 32 mg/L. Each strain shows the cell length (vertical axis) in, from left to right (horizontal axis), control cultures (0), cultures incubated with 0.5-mg/L mitomycin C (MMC) and cultures incubated with increasing doses of cotrimoxazole alone, each paired with incubation of cotrimoxazole together with 0.5-mg/L MMC.

Three strains of *E. coli* and three of *K. pneumoniae* with increasing MICs of cotrimoxazole were incubated with increasing concentrations of the antibiotic, ranging from 0.06 to 32 mg/L, to evaluate both (1): the cotrimoxazole-induced lengthening (Figure 2) and (2) cotrimoxazole-induced suppression of MMC-linked elongation (Figure 3 and Figure 4). Given that the CLSI breakpoint concentration of susceptibility is 2/38 mg/L trimethoprim/sulfamethoxazole, two *E. coli* strains were categorized as susceptible (MICs 0.125 and 0.38 mg/L, respectively) and one resistant (MIC > 32 mg/L). Regarding **K. pneumoniae**, one was susceptible (MIC: 0.38 mg/L), one was at the susceptibility breakpoint (MIC: 2 mg/L) and one was resistant (MIC > 32/mg/L).

(1) Cotrimoxazole induced statistically significant lengthening with respect to each respective control untreated culture when reaching a concentration similar or close to the MIC of the strain (Figure 2). The median length may increase even more with subsequent doses, but this was not statistically significant, thus remaining stable at the highest doses, although with a lower variability. No elongation was evidenced in the resistant strains (MIC > 32 mg/L) at any dose (Figure 2). (2) Regarding MMC-linked elongation, a decrease in the MMC-induced extra lengthening was evident at concentrations of cotrimoxazole close or similar to the MIC, so that the bacterial length was not significantly different to that produced by cotrimoxazole alone (Figure 3 and Figure 4). This absence of differences was maintained at higher doses. The resistant strains (MIC > 32 mg/L) never showed a decrease of cell length of MMC at any dose of cotrimoxazole assayed (Figure 3 and Figure 4).

Given that, according to the dose response, it is evidenced that the effect is significant at or close to the MIC of the strain, the incubation with the CLSI breakpoint concentration of susceptibility based on the criterion of cell growth inhibition, i.e., 2/38-mg/L trimethoprim/sulfamethoxazole, was chosen to be validated for the discrimination of susceptibility or not based on the two cell elongation assays.

### 2.3. Assay Validation

Clinical isolates of *E. coli* and **K. pneumoniae** were blindly processed following the protocol of the assay. Both parameters: (1) cotrimoxazole-induced lengthening and (2) cotrimoxazole-induced suppression of MMC-linked extra elongation were estimated and compared with the results of the standard antibiogram (Table 1). (1) Cotrimoxazole-induced lengthening resulted in five false negatives of the identification of resistance, two in *E. coli* and three in **K. pneumoniae**. These strains enlarged despite being non-susceptible, and a cotrimoxazole prescription would be ineffective, with threatening consequences in cases of severe infection. Moreover, four false positives of resistance were identified, one for *E. coli* and three for **K. pneumoniae**. These strains did not enlarge despite being susceptible but were not clinically relevant, since another effective antibiotic was selected. (2) Importantly, neither false positives nor false negatives were evidenced when the parameters of the cotrimoxazole-induced suppression of MMC-linked elongation were used; all the strains were correctly identified.

## 3. Discussion

MMC mainly induces DNA interstrand crosslinks that trigger the bacterial SOS response, a complex regulatory network for DNA repair [15,16]. The response is possible through the short-term expression of inducible proteins. One of these proteins is SulA (or SfiA), which is an inhibitor of the protein FtsZ. The latter is necessary to constitute the contractile ring in the future septum required for cell division [14,17]. As a result, cytokinesis is prevented to allow DNA repair before segregation. However, the cell continues to grow, and the morphology lengthens [18]. In fact, given its effectiveness, MMC is commonly employed as a positive control in experiments dealing with the SOS response, including the cell elongation effect [19,20].

Overall, the SOS response requires a rapid RNA transcription and coupled ribosomal protein synthesis [21]. In a previous report, it was demonstrated that the antibiotic inhibitors of protein synthesis prevented MMC-SOS linked cell elongation when the strain was susceptible to the antibiotic [12]. Possibly, the general inhibition of the protein synthesis at the ribosomes by the antibiotic included the SOS-inducible SuIA. Nevertheless, in resistant strains, the antibiotic could not be effective in inhibiting protein synthesis, and therefore, the MMC-induced cell lengthening was maintained. The optimization of the procedure allowed a simple and rapid assay to determine the susceptibility or resistance to the antibiotic inhibitors of protein synthesis in **E. coli*, *K. pneumoniae*, Acinetobacter baumannii* and *Pseudomonas aeruginosa* [12].

The essence of the assay is the prevention or nonprevention of the synthesis of the proteins necessary for cell division, like SuIA. However, this prevention could take place not only in the final step of protein synthesis in the ribosome but, also, in the previous step of RNA transcription. Cotrimoxazole produces a sequential inhibition of the enzymes of the tetrahydrofolate synthesis pathway. The depletion of tetrahydrofolate impedes the synthesis of thymidine essential for DNA replication, the synthesis of purines required for DNA replication and RNA transcription and the synthesis of methionine necessary for protein synthesis. Although the influence of the other physiological processes cannot be discarded, the deficiency of purines would affect the RNA transcription necessary for some of the outcomes of the induced SOS response, including the inhibition of bacterial division.

Intriguingly, trimethoprim and cotrimoxazole induce bacterial enlargement in the susceptible strains of many Enterobacteriaceae, and the induction of the SOS response has been suggested as responsible, as with the MMC [11,22]. It seems paradoxical that trimethoprim, an inducer of bacterial enlargement, prevents extra bacterial lengthening by the MMC. When combined cotrimoxazole and MMC, on one side, the bacteria is enlarged by cotrimoxazole, but on the other side, the bacteria cannot go on enlarging at the level achieved by MMC alone. Nevertheless, this response is not universal, and some susceptible strains do not enlarge with trimethoprim but show lengthening after incubation with MMC [11]. In fact, our study identified four strains that did not enlarge after cotrimoxazole despite being susceptible, i.e., false positives, according to this parameter. Nevertheless, these strains were enlarged by MMC, and cotrimoxazole prevented this MMC-induced lengthening. Although trimethoprim may induce a SOS response, this differential behavior suggests that the SOS response is questionable as the main factor responsible for cell elongation by this antibiotic. Alternatively, the impaired RNA transcription due to deficiency of purines may compromise the ribosomal translation, resulting in the depletion of some proteins necessary for bacterial division, so the cell lengthens. Otherwise, this same mechanism could impede the extra lengthening linked to SOS when MMC is later added to the culture, as previously discussed.

In the previous report about cell lengthening by trimethoprim, the effect was evaluated after 4 to 5 h and 16 h of incubation [11]. Although some more species from *Enterobacteriaceae* were tested, only very scarce strains were scored, e.g., 14 *E. coli* strains, including six resistant and three susceptible *K. pneumonia* strains. Here, we optimized the procedure for cotrimoxazole in *E. coli* and **K. pneumoniae**, obtaining the results in 2 h and 45 min and testing many more strains. Although the induced lengthening was revealed as a reliable parameter for the discrimination of susceptibility or resistance to cotrimoxazole, the accuracy of the evaluation of the induced suppression of MMC-linked elongation was complete in our clinical samples, demonstrating its superiority.

This is a rapid and simple phenotypic assay that detects resistance without the need to previously know the causal mechanism, as usually required when using molecular genetics or proteomic procedures. Evaluation of the cell length is a simple parameter that may be easily determined with a standard automatized microscopic or flow cytometry platforms. The present work elucidated that the evaluation of the prevention or nonprevention of MMC-induced lengthening, successfully applied for the rapid determination of resistance to antibiotic inhibitors of protein synthesis, may also be extended to cotrimoxazole, thus helping to preserve the newer broad-spectrum antibiotics and last option antibacterial drugs.

## 4. Materials and Methods

### 4.1. Bacterial Strains and Minimum Inhibitory Concentrations (MICs) Determination

Clinical isolates of *E. coli* (*n* = 50) and **K. pneumoniae** (*n* = 52) were collected at the University Hospital A Coruña between 2001 and 2018. MICs to cotrimoxazole were ascertained by automated microdilution (MicroScan Walkaway, Siemens, Munich, Germany) and corroborated by a Liofilmchem MIC Test Strip according to the manufacturer’s instructions, with susceptibility defined using CLSI criteria [23].

### 4.2. Determining Susceptibility-Resistance Using Cell Elongation

Bacteria cultured on solid media plates were used to inoculate a 2-mL Mueller–Hinton broth culture and incubated at 37 °C for 90 min. Afterwards, the bacteria were diluted to an OD_600_ of 0.1 in Mueller–Hinton broth and incubated at 37 °C in 200-μL tubes. Cotrimoxazole (6 μL) was added to the culture 30 min before the addition of the MMC (0.5 mg/L), and both were maintained for 2 h of incubation in a final volume of 30 μL. The concentration and incubation time of MMC was optimized previously in *E. coli* and **K. pneumoniae** [10], corresponding to the minimum dose and time that provided the highest lengthening without decreasing the cell number.

In order to assess the cell length, the bacteria were diluted to a concentration of 1 × 107 microorganisms/mL in Mueller–Hinton broth, included in a thin agarose microgel on a slide, dehydrated in increasing ethanol baths, dried, stained with the fluorochrome SYBR Gold and examined under an epifluorescence microscope (Nikon E800). Images were captured using a high-sensitive CCD camera (KX32ME, Apogee Instruments, Roseville, CA, USA). Image analysis was performed using NIS-Elements software (Nikon Instruments Inc., Tokyo, Japan), measuring the length of 100 cells per assay.

Cultures with neither cotrimoxazole nor MMC, with cotrimoxazole only and with MMC only were always processed simultaneously. Controls without antibiotic and MMC provided the basal cell length of each specific strain. The culture with MMC established the elongation size. The culture with cotrimoxazole co-incubated with MMC revealed if there was prevention or not of MMC-induced cell enlargement.

The relationship between the prevention of MMC-induced cell elongation and the CLSI breakpoint concentrations of cotrimoxazole were empirically established after processing a subset of strains comprising a wide range of MICs, including susceptible and resistant strains exposed to progressively increasing antibiotic doses. Several strains with MICs close to the CLSI breakpoints for susceptibility and resistance were employed. Antibiotic concentrations that suppressed MMC-induced cell enlargement in the susceptible, but not in the resistant strains, were determined. It should be noted that the suppression of MMC-induced cell elongation was considered when the bacterial size was not significantly different from that of the cotrimoxazole-only treated culture.

The concentrations of antibiotics were optimized such that a strain categorized as susceptible following the CLSI criteria demonstrated the abolition of cellular elongation after incubation with the lowest concentration of antibiotic established as the susceptibility breakpoint when using the parameter of elongation suppression. Notably, a non-susceptible strain should not show any abolition of MMC-induced elongation with the established breakpoint concentration of the antibiotic.

Once implemented, the breakpoint concentration for the elongation suppression criterion, which correlates with the CLSI susceptibility breakpoint for each antibiotic and bacterium, the collected isolates were blindly processed. For each isolate, four cultures were established, as indicated above: (1) a control culture with neither cotrimoxazole nor MMC, (2) a culture with the established breakpoint dose of antibiotic only, (3) a culture with MMC only and (4) a culture with cotrimoxazole (the established breakpoint dose) and MMC.

### 4.3. Data Analysis

Data were analyzed using the SPSS Statistics 21 software package for Windows (IBM). Friedman test and Wilcoxon test with Bonferroni correction were employed for statistical comparisons. Significance was defined as *p* < 0.05. Receiver operating characteristic (ROC) curves were constructed with the results obtained during the validation assay. The area under the curve (AUC) values indicated the global classification ability of the test. Validation indexes were estimated using the Epidat 3.1 software package (Consellería de Sanidade, Xunta de Galicia, Spain and Panamerican Health Organization, Washington, DC, USA).

## Figures and Tables

**Table 1:** Analytical evaluation of the technical assays of cotrimoxazole-induced cell elongation (CTX) and cotrimoxazole prevention of mitomycin C-induced cell enlargement (CTX+MMC), in comparison with the categorization by the standard antibiogram and CLSI criteria of Escherichia coli and Klebsiella pneumoniae strains. S: susceptible; NS: non-susceptible; AUC: area under the receiver operating characteristic (ROC) curve.

Microorganism	CLSI Classification		Technical Assay	Sensitivity (%), 95% CI	Specificity (%), 95% CI	AUC, 95% CI, *p* < 0.0001
	S	NS				
*E. coli*	23	27	CTX	92.59 (80.86–100)	95.65 (85.14–100)	0.941 (0.866–1.00)
			CTX+MMC	100 (98.15–100)	100 (97.83–100)	1 (1.00–1.00)
*K. pneumoniae*	34	18	CTX	83.33 (63.34–100)	91.18 (80.17–100)	0.873 (0.757–0.988)
			CTX+MMC	100 (97.22–100)	100 (98.53–100)	1 (1.00–1.00)

## Data Availability

Not Applicable.
